# Improved support vector machine algorithm based on the influence of Gestational Diabetes Mellitus on the outcome of perinatal outcome by ultrasound imaging

**DOI:** 10.12669/pjms.37.6-WIT.4855

**Published:** 2021

**Authors:** Hehua Liu, Jie Liu

**Affiliations:** 1 Hehua Liu, Attending Physician. Department of Obstetrics, East Hospital of The Fourth Hospital, Shijiazhuang City, 050011, China; 2 Jie Liu, Attending Physician. Department of Emergency Medicine, East Hospital of The Fourth Hospital, Shijiazhuang City, 050011, China

**Keywords:** Gestational diabetes, Pregnancy complications, Umbilical artery, Middle cerebral artery, Uterine artery, Ultrasound examination

## Abstract

**Objectives:**

In order to understand the incidence and epidemiological characteristics of gestational diabetes mellitus, the ultrasound imaging of support vector machine processing algorithm was used to clarify the outcome of maternal and neonatal gestational diabetes mellitus.

**Methods:**

This study selected clinical data of 12,190 pregnant women who were hospitalized for delivery, and were divided into diabetic group (1268 cases) and control group (10922 cases) according to the diagnosis of gestational diabetes. The study was conducted from January 1, 2012 to December 31, 2019. Colour Doppler ultrasound was performed to record fatal umbilical artery and brain the middle arteries and uterine arteries which are effective indicators of measuring fatal intrauterine conditions. Chi-square test was used to compare the rates between groups, and multivariate logistic regression was used for labour outcomes.

**Results:**

The incidence of diabetes during pregnancy is about 10.4% (1268/12190). Senior citizens and women suffering from obesity increase the risk of gestational diabetes, maternal hypertension disorders in pregnancy, premature rupture of membranes, oligohydramnios, fatal distress, multiple births, malpresentation risk increased significantly (P <0.05) than the control group. In gestational diabetes caesarean section rate was significantly higher (61.0% vs46.4%). Caesarean new born 5-minute Apgar score was significantly lower than the control group (P <0.05).

**Conclusion:**

In maternal gestational diabetes in high risk pregnancies, complications of pregnancy significantly increase the importance of enhancing weight management and blood glucose monitoring to reduce complications.

## INTRODUCTION

Previous studies have shown that the risk of pregnancy complications of GDM pregnant women, such as premature birth, giant infants, and hypertension during pregnancy, is significantly increased.^1^-^3^ The increase in the incidence of large size infants also increases the rate of caesarean section. Complications also increased significantly, manifested as hypoglycaemia, respiratory distress syndrome, jaundice, fatal growth restriction, and increased morbidity of children less than gestational age, which resulted in a significant increase in the rate of neonatal conversion to ICU.^4^-^7^ Therefore, the diagnosis of GDM and the monitoring and management of blood glucose have a great impact on pregnancy outcomes.^8^,^9^ At present, there are not many cross-sectional studies on the perinatal outcome of GDM in China.^10^,^11^ This study collected the data of hospitalized women who delivered in the hospital, and analyzed the data to understand the incidence of GDM in the hospital under the new GDM diagnostic standard. We also studied the related maternal complications, maternal and neonatal perinatal outcomes.

## METHODS

We collected clinical data of all hospitalized deliveries from January 1, 2012 to December 31, 2019 patients and puerperal ≥ 24 weeks of gestation, including general conditions such as age, education level, and pregnancy times, delivery methods and complications and the condition of the new born. All the clinical data were collected from medical records and the study was approved by the hospital ethics committee.

A total of 12,190 patients clinical data were collected and divided into a GDM group (1268 cases) and a control group (10922 cases) according to whether GDM was diagnosed. GDM diagnosis was based on the 2010 IADPSG to develop new diagnostic criteria: all non-diabetic pregnant women routinely undergo 75g oral glucose tolerance test (OGTT) at 24 to 28 weeks of gestation, meeting or exceeding at least one of the following indicators: fasting blood glucose, after taking sugar 1 The 2-hour blood glucose was 5.1 mmol/L, 10.0 mmol/L and 8.5 mmol/L, respectively, which was the diagnosis of GDM. Prenatal haemorrhage includes placenta previa, placental abruption, and rupture of blood sinus at the edge of placenta. Pre-exposed abnormality refers to pre-exposed pre-existing abnormalities including breech and lateral position, excluding occipital abnormalities at birth, such as persistent lateral and posterior occipital positions.^12^,^13^

Ultrasound examination. Using E8 color ultrasound Doppler ultrasound diagnostic equipment from the US company GE, the probe frequency is 2.5 to 3.5 MHz Obstetrical conditions were adopted. UMA, MCA and UTA were tested after routine obstetrical examination. The sound beam was parallel to the direction of blood flow as far as possible. The correction angle was less than 20°. The pulse Doppler sampling volume was selected according to the thickness of the vessel diameter. The 3mm measurement was selected for the MCA measurement. When testing UMA, choose a sample within 5-cm of the place where the placenta is inserted, adjust the position and angle of the probe so that the sound beam is parallel to the direction of blood flow. After obtaining a stable spectrum image, the blood flow parameters are measured by stopping the frame. Of the foetus through the UTA and UMA, MCA flow spectrum is measured separately collecting the blood vessel and the following recording parameters: Pulsatilityindex (PI), Resistance in- deck (RI), systolic flow / diastolic flow (S / D). The perinatal follow-up content includes the birth method, the fatal perinatal outcome of the new born 1minAgar score, birth weight, neonatal admission to the NICU ward and mortality rate, all data were tabulated and statistically analyzed.

### Statistical Analysis

After all the data are entered into the computer to establish a database for statistical analysis was done using SPSS18.0. Normally distributed data were expressed as mean ± standard deviation, skewness distribution data were median and interquartile. Continuous variables were skewed using Mann-Whitney U analysis, χ^2^ test was used to compare the frequency. SVM is the optimal classification surface in linear separable situation evolved. The classification line equation is, and we can normalize it to make the linearly separable sample set B.


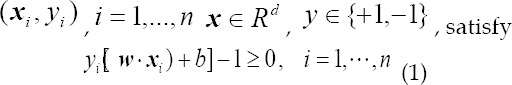



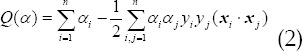



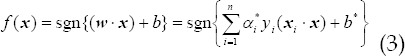


The summation in the formula is actually only performed on the support vector. b * is the classification threshold, which can be obtained by any one of the support vectors (satisfying the equal sign in (1)), or by the median of any pair of support vectors in the two categories.


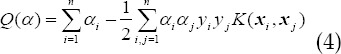


And the corresponding classification function also becomes


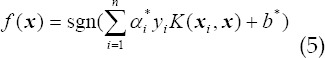


This is the support vector machine. If you want to find a certain balance between empirical risk and generalization performance, you can allow the existence of misclassified samples by introducing a positive relaxation factor ξi. At this time, the constraint (1) becomes


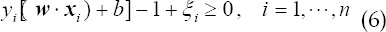


And the penalty term 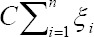 is added to the goal-minimizing 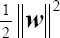 that the Wolf dual problem can be written as:


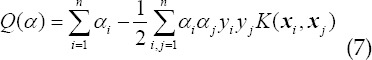


## RESULTS

The study included 12,190 pregnant women, women with GDM 1268 cases, the incidence being 10.4%. The average age of GDM women (31 years old) was significantly higher (30, 1) (P <0.001); GDM group mean gestational age (38 years) was significantly less than the control group (39) (P <0.001). GDM maternal obesity, pregnancy induced hypertension, premature rupture of membranes, oligohydramnios, fatal distress, oligohydramnios, multiple pregnancy, the risk malpresentation than the control group increased significantly (P <0.05). GDM mother’s adverse pregnancy history, premature birth, fetal growth restriction, prenatal risk of haemorrhage than the control group, the difference was not significant (Table-I).

**Table-I:** Study population characteristics [n (%)].

*Project*	*GDM group*	*Control group*	*P*
History of poor pregnancy	31 (2.4)	185 (1.7)	> 0.05
obesity	366 (31.0)	1897 (18.2)	<0.01
Multiple births	331 (2.6)	192 (1.8)	<0.05
Premature delivery	92 (7.3)	702 (6.4)	> 0.05
Premature rupture of membranes	306 (24.1)	2929 (26.8)	<0.05
Hypertension during pregnancy	126 (9.9)	590 (5.4)	<0.01
Fatal growth restriction	29 (2.3)	307 (2.8)	> 0.05
Prenatal bleeding	33 (2.6)	232 (2.1)	> 0.05
Abnormal	89 (7.0)	606 (5.5)	<0.05
Too little amniotic fluid	32 (2.5)	422 (3.9)	<0.05
Fatal distress	202 (15.9)	2545 (23.3)	<0.01

*Note:* Missing data; 30 cases of adverse pregnancy history, 484 obese patients, and 90 cases of fatal growth restriction.

As regards characteristics of GDM maternal pregnancy outcomes. GDM mothers’ children weight was significantly higher (P <0.05). Comparison of neonatal sex postpartum haemorrhage and maternal morbidity two groups showed that the difference was not statistically significant. 1,269 cases of maternal GDM, caesarean delivery 773 cases (61.0%), compared with the control group (46.4%) was significantly higher; natural vaginal delivery 466 cases (36.8%), vaginal delivery 29 cases (2.3%), compared with the control group decreased significantly. Compared with the control group, the 1-minute Apgar score of GDM maternal neonates was less than 7 (0.2%), and there was no significant difference, while 13 cases (1.0%) of 5-minute Apgar score was less than 7 (Table-II).

**Table-II:** Comparison of the delivery outcomes of the two groups.

*Project*	*GDM group*	*Control group*	*P*
Huge	130 (10.3)	720 (6.6)	< 0.01
Male tire	692 (54.6)	5701 (52.2)	> 0.05
Vaginal delivery	466 (36.8)	5429 (49.7)	< 0.01
Surgical delivery	29 (2.3)	425 (3.9)	< 0.01
Caesarean section	773 (61.0)	5068 (46.4)	< 0.01
Apgar score <7 in 1 minute	3 (0.2)	23 (0.2)	> 0.05
Apgar score <7 in 5 minutes	13 (1.0)	1 (0.0)	< 0.01
Postpartum haemorrhage	116 (9.1)	929 (8.5)	> 0.05

*Note:* Missing data: 71 cases of fatal weight, 68 cases of Apgar score in 1 minute, and 125 cases of Apgar score in 5 minutes.

There are two sets of indications for caesarean section analysis. In order to clarify the reason for the significant increase in the cesarean section rate in the GDM group, the study further compared the two groups of cesarean section indications. The results showed that the rate of diabetic cesarean section without medical indications was significantly higher than that of the diabetic group without medical indications. Obese women who had a Caesarean section accounted for 37.1% (56/151), which was significantly higher (21.4%, 204/952). Secondly, the characteristics of diabetic uterine scars, elderly women, older children, preeclampsia, multiple pregnancy, cesarean section, etc. were also significantly increased (P<0.05) (Table- 3).

**Table-III:** Comparison of indications for caesarean section [n (%).

*Caesarean section indications*	*Control group*	*GDM group*	*P*
No indication	994 (9.1)	161 (12.7)	< 0.01
Fatal distress	717 (6.6)	70 (5.5)	> 0.05
Stagnation of labour	593 (5.4)	75 (5.9)	> 0.05
Hip, horizontal position	540 (4.9)	78 (6.2)	> 0.05
Scarred uterus	404 (3.7)	67 (5.3)	< 0.01
Elderly maternal	399 (3.7)	114 (9.0)	< 0.01
Huge	359 (3.3)	65 (5.1)	< 0.01
Preeclampsia	232 (2.1)	49 (3.9)	< 0.01
Multiple births	157 (1.4)	30 (2.4)	< 0.05
Prenatal bleeding	143 (1.3)	18 (1.4)	> 0.05

Comparison of Doppler ultrasound results showed that the study group uterine artery PI, RI had no statistically significant difference compared with control group (P> 0.05), Table IV-VI.

**Table-IV:** PI, RI, S/D comparison of fatal umbilical artery.

	*PI*	*RI*	*S/D*
Research group	1.34 ± 0.33	0.68 ± 0.05	3.70 ± 0.87
Control group	0.76 ± 0.13	0.59 ± 0.06	2.45 ± 0.42
t	12.252	8.167	9.627
P	<0.05	<0.05	<0.05

**Table-V:** PI, RI, S / D comparison of fatal middle cerebral artery in two groups.

	*PI*	*RI*	*S/D*
Research group	1.09 ± 0.36	0.64 ± 0.17	3.22 ± 1.03
Control group	1.25 ± 0.34	0.76 ± 0.29	3.95 ± 0.96
t	2.322	2.49	3.729
P	<0.05	<0.05	<0.05

**Table-VI:** PI, RI, S / D comparison of two groups of uterine arteries.

	*PI*	*RI*	*S/D*
Research group	0.60 ± 0.17	0.43 ± 0.09	1.88 ± 0.06
Control group	0.55 ± 0.13	0.42 ± 0.08	1.71 ± 0.22
t	1.701	0.599	5.088
P	>0.05	>0.05	<0.05

Various pregnancy comorbidities can cause spasm of uterine myometrium and decidual layer arteries, thickening of the tube wall, reduction of the inner diameter of the uterine cavity. Decreased blood flow is manifested by increased S/D of the umbilical artery. The increased resistance of the placental circulation directly affects the exchange of nutrients and oxygen between the fatal circulation and maternal blood, resulting in different degrees of intrauterine hypoxia in the foetus. The statistical significance (P<0.05) suggests that the combined application of umbilical artery blood flow parameters to predict fatal intrauterine status has a high clinical value.

## DISCUSSION

In this study, the caesarean section rate of gestational diabetes was significantly higher, reaching 61%. Obese elderly women have an increased risk of gestational diabetes, which may lead to maternal hypertension, premature rupture of membranes, oligohydramnios, and fatal distress.^14^-^16^

Obesity during pregnancy is a high risk factor for GDM. In this study, the perinatal outcome of GDM women was analyzed.^17^,^18^ Previous studies have shown that, age, high BMI, excessive weight during pregnancy, and high blood pressure are risk factors for GDM. Pinheiro 19-20 have shown that, older woman (older than 35 years old) are more likely to be overweight and suffer from gestational diabetes and high blood pressure, and they prefer to have caesarean section, with poor perinatal period, which is consistent with the results of this study. The perinatal mortality rate of older women is higher. In the present study, the gestational week of GDM women was significantly lower than that of the control group. This may be why the guidelines recommend GDM women to induce labor at 40 weeks.

## CONCLUSIONS

Studies have shown that there is a statistical difference between low birth weight infants and neonates in the control group and the low group study. There were neonatal deaths in the study Group-2 and no neonatal deaths in the control group. It also showed that the difference between the study group and the control group in perinatal adverse outcomes was statistically significant, and the abnormal Doppler spectrum is significantly correlated with the adverse perinatal outcomes. As such the three-vascular Doppler spectrum hemodynamic examination is closely related to predicting adverse perinatal outcomes and reducing pregnancy risks.

### Authors Contribution:

**HL:** Conceived the study, literature review, drafting the manuscript, data analysis,

**JL:** Takes the responsibility and is accountable for all aspects of the work in ensuring that questions related to the accuracy or integrity of any part of the work are appropriately investigated and resolved.
